# A Case with late onset of ambiguous genitalia

**Published:** 2017-03

**Authors:** Soraya Saleh Gargari, Faezeh Azizi, Nasrin Saleh, Mir Davood Omrani

**Affiliations:** 1 *Feto-Maternal Unit, Mahdieh Hospital, Shahid Beheshti, University of Medical Sciences, Tehran, Iran. *; 2 *Department of Medical Genetics, Faculty of Medicine, Shahid Beheshti University of Medical Sciences, Tehran, Iran. *; 3 *ICU Unit, Shaheed Rajaei Cardiovascular Medical and Research Center, Tehran, Iran.*

**Keywords:** Ambiguous genitalia, Male pseudohermaphroditism, Mutations

## Abstract

**Background:**

Ambiguous genitalia is an uncommon situation that happens between 1 and 2 per every 1000 live births and falls under the umbrella diagnosis of disorders of sexual development.

**Case:**

In this article, we report a case of male pseudohermaphroditism with ambiguous genitalia. The proband was a 12 yr old girl without any uterus or ovarian tissues. Karyotype of the case is 46, XY. Genes involved in sexual differentiation such as AR, SRD5A2, LH, LHR, FSH, 17 B HSD and SRY genes were sequenced in both directions. No mutations were found in these genes either.

**Conclusion:**

It seems advisable to be cautious in similar cases, and revise protocol for tracing the genes involved in the patients.

## Introduction

Ambiguous genitalia is a birth fault where the outer genitals do not have the classic aspect of either a girl or a boy. The disturbances of sexual development 46,XY are scarce, and the most frequent etiology is androgen insensitivity (1, 2). “Copy number variations or mutations of several sex determining genes, including but not limited to SRY, NR5A1/SF1, DAX1, WT1, SOX9, and GATA4, result in gonadal streaks or dysgenetic testes in people with a 46,XY karyotype” (3). Because most people with 46,XY DSD do not have any of these identified mutations, more genes requirement for development of a bipotential gonad into following testicular differentiation are determined to be known with time (4-7). 

## Case report

**Clinical Description**

The patient was a 12-years old girl of non-consanguineous Iranian parents with an ambiguous genitalia and 46,XY karyotype. Gestation was normal and at birth the patient had no dysmorphic feature. No history of birth defects, and congenital malformations were seen in the family. This patient had normal female external genitalia at birth. During pubertal development secondary male sexual characteristics were noticed. Her histopathology results showed a small testis covered by tunica albuginea and fibro-connective tissue measuring 1.8x0.9x0.9 cm, epididymis 1.3x1.2x0.7 cm and vasodefrens measuring 4.5 cm in length and 1 cm in diameter.

Sections showed fibrous tissue and dilated congested vessels. Seminiferous tubules with the presence of Sertoli cells and hyalinization of them (immature testis) were seen. No uterus or ovarian tissue was identified. Serum FSH, and LH were in normal range but her serum testosterone level was high (1.2 ng/ml). Sampling for this project was down in Iran but cytogenetic and sequencing was performed in molecular medicine of Karolinska Institutet -a medical university in Sweden from April to June 2015.

**Cytogenetic and Molecular Analysis**

**Figure 1:**
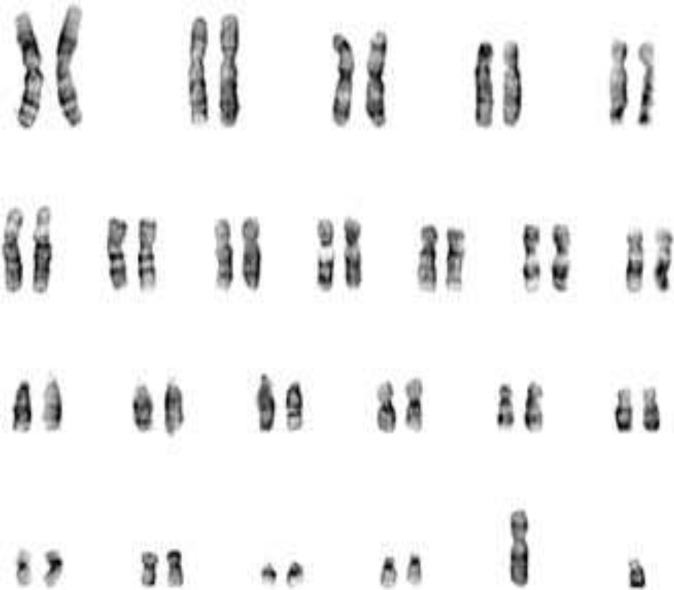
46, XY karyotype in a case with female phenotype

**Figure 2.**
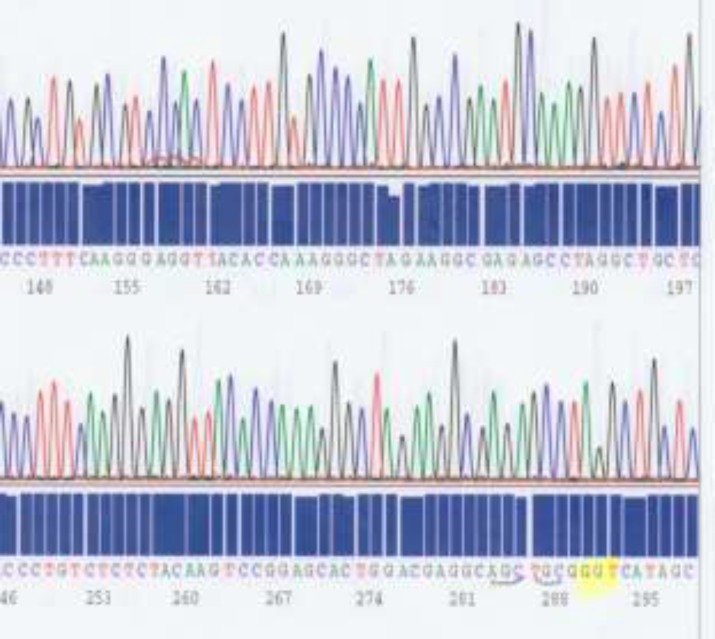
A part of chromatograph patterns with intact *AR* gene sequences in our patient

SRY gene was designed using the web- based primer-blast program by standard selection criteria. Polymerase Chain Reaction (PCR) reactions were performed with (Roche Diagnostics, Mannheim, Germany) using the standard protocol. Genes involved in the process of sex determination such as AR, LH receptor, 5 alfa reductase (SRD5A2), 17 B HSD, and SRY gene, were sequenced and analyzed. GTG banded karyotype of the patient showed 46, XY (Figure 1). No chromosomal abnormalities were noticed in her parents. Regarding molecular analysis by sequencing method (Figure 2), no mutation identified among AR, SRD5A2, LH, 17 B HSD and SRY gene. After consultation and interview with patient and her parents and reaching to conclusion for her future sexual identity, she underwent left orchidectomy and then vaginoplasty male to female procedure. Follow-up after 5 years showed that her psychosocial orientation was geared towards female gender. She dressed up and identified herself as the female in the community.

**Ethical consideration**

The patient has provided written consent for the case report to be published.

## Discussion

There is still much to learn about DSD disturbances, this documented by the fact that, although the many processes of genitalia development is under intense research, many directions of sex determination and differentiation in the 46,XX and 46,XY individuals remain unknown (8). The term male pseudohermaphrodite is applied when a testis is existent (9). Mutations affecting the AR gene may reason either partial or complete androgen insensitivity syndrome (PAIS and CAIS). Androgen, a hormone used to define a category of sex steroid hormones, is accountable for affecting male pseudohermaphroditism. The differentiation of the fetus as male happens during the 6th or 7th weeks of gestation. The development is controlled by the SRY gene. after 9th-13th weeks, the development of a male genitalia is contingent the shift of testosterone to the more potent androgen by the function of 5α-reductase within the genitalia (10).

Persistent Müllerian duct syndrome is a type of internal male pseudohermaphroditism, which is developed via synthesis of Müllerian-inhibiting factor deficiencies. In such models, duct derivatives are now in 46XY males-this includes the upper vagina, uterus, and fallopian tubes. These persons with a hernia sac and bowel loops were found with duct derivatives also testes (11). “The patient reported herein represents sporadic and incomplete form of the XY gonadal dysgenesis syndrome” (12). 

This case had normal female external genitalia at birth. During pubertal development secondary male sexual characteristics were noticed. On investigation manifested no Mullerian structures. The dysgenetic testes were capable of producing an enough amount of anti-Mullerian hormone for Mullerian regression and testosterone for Wolffian development, although they were incapable of producing enough amount of testosterone for complete masculinisation of external genitalia. “One of the many challenges to understanding the natural history of DSD in people with 46,XY is that, these conditions are heterogenous in nature and only a portion of cases can be attributed to known genetic causes at this time” (3). 

In this research, we checked the most important genes as the standard protocol says for sex determination in similar cases, but no mutations were identified. So, it seems, due to limitations in the current protocol instructions to identify the causes of disease, it is necessary to review and upgrade diagnostic protocols of DSDs. New technologies for perception the multiple etiologies of 46,XY DSD, such as array sequencing, array comparative genomic hybridization, and next generation sequencing may result in a super understanding of the genetic causes of DSD for patients who possess a Y chromosome. Albeit, these improvements and developments have not yet been implemented clinically (13). 
